# Post-Mortem Detection and Visualization of Mimivirus Reactivation in Fatal Viral Pneumonia

**DOI:** 10.3390/v18030379

**Published:** 2026-03-18

**Authors:** Parandzem Khachatryan, Naira Karalyan, Anna Semerjyan, Marina Tatoyan, Hakob Davtyan, Arsham Yeremyan, Sona Hakobyan, Hranush Avagyan, Lina Hakobyan, Liana Abroyan, Aida Avetisyan, Elena Karalova, Nane Bayramyan, Tigranuhi Vardanyan, Vahagn Gevorgyan, Elina Arakelova, Alexandr Karalyan, Zaven Karalyan

**Affiliations:** 1 Department of Pathology, Yerevan State Medical University After Mkhitar Heratsi, Yerevan 0025, Armenia; 2 Department of Pathological Anatomy and Clinical Pathology, Yerevan State Medical University After Mkhitar Heratsi, Yerevan 0025, Armenia; 3 Department of Medical Biology, Yerevan State Medical University After Mkhitar Heratsi, Yerevan 0025, Armenia; 4 Department of Histology, Yerevan State Medical University After Mkhitar Heratsi, Yerevan 0025, Armenia; 5 CANDLE Synchrotron Research Institute, 31 Acharyan Str., Yerevan 0022, Armenia; 6 Laboratory of Cell Biology and Virology, Institute of Molecular Biology of NAS RA, Yerevan 0014, Armenia; 7 Viral Ecology Research Group, Institute of Molecular Biology of National Academy of Sciences of the Republic of Armenia, Ezras. Hasratyan 7, Yerevan 0014, Armenia; 8 Experimental Laboratory, Yerevan State Medical University After Mkhitar Heratsi, Yerevan 0025, Armenia; 9 Scientific Center for Risk Assessment and Analysis in Food Safety Area, 107/2 Masis Highway, Yerevan 0071, Armenia

**Keywords:** mimivirus, mimiviridae, viral pneumonia, NCLDV, post-mortem pathology, alveolar macrophages, host–pathogen interaction, lung tissue

## Abstract

Mimivirus, a giant double-stranded DNA virus ($1.2$ Mbp), possesses unique bacteria-like features, including a Gram-positive staining reaction due to peptidoglycan-containing surface fibers. While detected in the respiratory secretions of pneumonia patients since 2005, its clinical role remains controversial due to high genetic variability and detection challenges. This study aims to clarify the pathological significance of Mimivirus by investigating its presence and replication potential in human lung tissue, specifically exploring its association with fatal respiratory outcomes. A comparative post-mortem analysis was conducted on lung tissue samples from two cohorts: patients who succumbed to lethal viral pneumonia and a control group with no history of pulmonary pathology. Mimivirus is known to productively infect alveolar macrophages, suggesting they may serve as a reservoir for lung inflammation and tissue damage. Current evidence suggests it may act as an opportunistic or commensal agent, particularly in immunocompromised or critically ill patients. By systematically screening autopsy samples, this research seeks to establish whether Mimivirus is a primary causative agent of fatal pneumonia or an incidental inhabitant of the human respiratory tract.

## 1. Introduction

Mimivirus, which stands for Microbe Mimicking Virus, is a massive virus that belongs to the Mimiviridae family. One of the biggest and most intricate viruses ever found, it was initially identified in 2003 [1]. At least several hundred genes are encoded by the massive double-stranded DNA genome (~1.2 million base pairs) of mimiviruses. Due to its size and Gram-staining capacity, it was initially thought to be a bacterium [2], but it is actually a DNA virus. Mimivirus has the genetic and morphological characteristics of a nucleocytoplasmic large DNA virus (NCLDV). The positive Gram-staining result is likely due to a component of the large virus [3]. They have fibrillar structures on the surface of their capsid, which are wrapped in long fibers composed of peptide glycan, a characteristic of bacteria. Other NCLDVs lack these fibers. The positive Gram stain result is likely due to this structural component of the large virus [3].

The potential clinical significance of Mimivirus in respiratory pathology remains a subject of ongoing investigation and debate. Research has identified Mimivirus DNA and, in some instances, viable viruses in respiratory secretions and bronchoalveolar lavage (BAL) fluid from patients with hospital-acquired and community-acquired pneumonia. Notably, studies employed molecular techniques such as real-time qPCR and viral isolation to screen cohorts of critically ill and mechanically ventilated patients. While these studies reported the presence of Mimivirus in a subset of fatal pneumonia cases, the scale of these investigations remains relatively small, and the degree of medical certainty regarding Mimivirus as a primary causative agent of pneumonia is not yet established. At present, distinguishing true clinical infection from transient colonization or potential false-positive results in molecular assays remains a significant technical challenge. Mimivirus-induced pneumonia in these patients can result in severe lung infection [4,5]. Additionally, positive Mimivirus serology was associated with prolonged ventilation times rather than increased mortality [6]. Mimivirus is generally rarely detected in the human lungs, especially in healthy individuals, and varies depending on the population and detection method [7]. However, it may be somewhat more common in patients with pneumonia or severe respiratory illness [8]. Multiple investigations attempting to detect mimiviruses in respiratory or environmental samples reported negative or very low positivity rates. These findings were initially interpreted as evidence of limited prevalence. However, subsequent genomic comparisons revealed that negative results were likely influenced by genetic polymorphism in primer target regions, rather than the true absence of the virus. As described in Raoult D, Levasseur, 2017, “In practice, the search for mimivirus is complicated by the great genetic variability of the virus and the restricted availability of mimivirus culture systems to a few research laboratories” [9].

Acanthamoeba polyphaga mimivirus can infect human and mouse macrophages via phagocytosis. Fibroblasts, lung epithelial cells, and other non-phagocytic cells are far less tolerant. Productive replication has also been demonstrated in human macrophages. According to Ghigo [10], this implies that alveolar macrophages could act as a reservoir, leading to lung inflammation, immunological activation, and damage.

Since the discovery of mimiviruses in human lungs in 2005 [8,11], studies have been conducted to determine whether mimiviruses are linked to or cause human disorders. The involvement of mimiviruses in human lung pathology has been studied for many years; however, there is insufficient scientific data to fully understand this issue. Furthermore, information on how frequently this virus is found in human lungs varies widely (see, for instance, [7,9,12]).

A mimivirus with a large virion size (~554 nm) and a genome larger than 1.2 Mb was discovered in 2013 in a lung sample from an elderly woman with pneumonia [4]. There have also been a few, however uncommon, reports of Mimivirus presence in different types of pneumonia. For instance, Mimivirus DNA was detected in sputum and bronchoalveolar lavage (BAL) samples from a 10-year-old boy with pneumonia in 2020 due to an underlying lung condition (primary ciliary dyskinesia) [13]. Mimivirus in human lungs is now regarded as an opportunistic or commensal virus that infrequently causes illness in immunocompromised individuals.

This research sought to detect Mimiviruses in human pulmonary tissue samples collected post-autopsy from two cohorts: patients with lethal viral pneumonia and those with no history of lung pathology at the time of death. This article aims to clarify the potential for viral replication in lung tissue and to explore the links between Mimivirus and fatal pneumonia in humans. Given the small number of fatal viral pneumonia cases with unknown etiology, and the even smaller number of cases of reliable mimivirus replication in human lungs, we do not claim to draw definitive conclusions regarding the role of mimivirus in lung pathology. The goal of this article is to identify possible associations between mimivirus and fatal pneumonia in humans, and to clarify the possibilities of viral replication in lung tissue.

## 2. Methods

### 2.1. Ethics Statement

The studies were reviewed and approved by the Ethics Committee of the Institute of Molecular Biology, NAS RA (IRB 00004079, 2013; Protocol N5 from 25 May 2018), Ethics Committee of Yerevan State Medical University after M. Heratsi (N12-9/25). The animal study protocol was approved by the Ethics Committee of the Institute of Molecular Biology, NAS RA (IRB 06042021/1, 2021).

### 2.2. Informed Consent Statement

Written informed consent was obtained from the patient(s) to publish this paper.

### 2.3. Human Lung Studies

The standard lung extraction procedure was used in each autopsy. Following ethical approval (Yerevan State Medical University; 12-9/25), we analyzed lung fragments (according to the ethics of Yerevan State Medical University (12-9/25) obtained from autopsy samples of four individuals who died between December 2024 and April 2025 with a diagnosis of viral pneumonia of various origins. The time interval between the individual’s death and the autopsy procedure was 6 h.

### 2.4. Lung Samples

Initially, the dead body was inspected macroscopically, the body cavities were opened, and the inner organs were evacuated. The organs were evaluated on an autopsy table; each lung tissue sample was measured and weighed. The sizes ranged from 146 to 193 cm, with a mean length of 173 cm. The weight ranged from 48.5 to 153 kg, with a mean weight of 76.4 kg.

At least five samples from different parts of the lung were taken from each case and fixed in 10% buffered formalin solution (pH 7.2) for 48 h. After fixation, the samples were dehydrated through a graded series of alcohols (70%, 80%, 96%, and 100%), washed with xylol to remove residual alcohols, and embedded in paraffin wax. For morphological analysis, wax-embedded samples were cut at 5 µm (Microm HM 355, Thermo Scientific, Waltham, MA, USA). Histological examinations were performed using a light microscope (BOECO, BM-800, equipped with a camera, B-CAM10, Boeco, Hamburg, Germany).

### 2.5. Staining Methods

Hematoxylin and eosin (H&E) staining was performed according to the standard procedure (Merck KGaA, Darmstadt, Germany) [14].

Lung sections stained with Van Gieson [15,16] were examined for vascular and interstitial disease. The percentage of collagen elements in the lungs was calculated by randomly selecting 100 visual fields from each patient and analyzing them in Photopea. Van Gieson-stained [15], lung sections were analyzed for interstitial and vascular pathology. From each patient, 100 visual fields were randomly selected and analyzed using Photopea software (Version 5.6) to determine the percentage of collagen elements in the lungs.

To visualize viral assemblies, we used the methyl green pyronin (MGP) (Merck KGaA, Darmstadt, Germany) method, one of the most convenient methods for detecting viral DNA structures in the cytoplasm of infected cells [17,18]. For the same purpose, the Feulgen–Naphthol Yellow (Merck KGaA, Darmstadt, Germany) protocol [19,20] was used.

To visualize viral particles, Gram staining (Merck KGaA, Darmstadt, Germany) of the preparations was performed according to the standard method [21], as modified by Lusi et al. [2].

### 2.6. Water Lysis of Samples with Mimiviruses from Human Lungs

To isolate mimiviruses, lysates were prepared by homogenizing human lung specimens (about 3.0 g) in double-distilled water, as described by [22,23,24]. Cellular debris and nuclei were then collected by centrifugation at 3000× *g* for 10 min.

### 2.7. Preparation of the Giant Viruses for Scanning Electron Microscopy (SEM)

Homogenized human lung specimens were frozen and thawed twice. The obtained lysates were centrifuged at 3000× *g* for 10 min at room temperature (~25 °C, RT) to remove cell debris. Homogenized tissue was resuspended in double-distilled water and centrifuged at 17,000× *g* for 5 min at 4 °C without a gradient [25]. Prior to SEM analysis, samples were sputter-coated with a thin conductive gold layer to minimize surface charging and improve secondary electron emission. Samples were mounted on aluminum stubs. The mounted specimens were placed in the sputter coater chamber and evacuated to a base pressure of approximately 0.05–0.1 mbar. High-purity argon gas (99.99%) was introduced as the sputtering medium to generate plasma. Gold deposition was carried out at a sputtering current of 20 mA under an argon atmosphere at a working pressure of ~0.1 mbar. The coating time was adjusted according to the calibrated deposition rate to obtain a uniform gold layer with an approximate thickness of 30 nm. Thickness control was monitored using the instrument’s built-in thickness control system.

### 2.8. High-Resolution Scanning Electron Microscopy

The sputter-coated samples of homogenized human lung specimens, after the above-mentioned procedures, were subjected to high-resolution electron imaging using a Carl Zeiss SEM EVO 10 (Carl Zeiss AG Vertriebs GmbH, Düsseldorf, Germany) to reveal virus particles in the human lungs.

### 2.9. Viral Nucleic Acid Extraction, Treatment with DNase, and cDNA Synthesis

Total viral RNA and DNA were extracted from pools of 20 adult males, females, or larvae, as well as pools of approximately 200 eggs, using the HiGene Viral RNA/DNA Prep Kit (BIOFACT, Yuseong-gu, Daejeon, Republic of Korea) according to the manufacturer’s instructions. All RNA/DNA samples were treated with DNase (Thermo Fisher Scientific Inc., Waltham, MA, USA) to ensure the removal of genomic DNA contamination for transcriptomic analysis. For gene transcription analysis, the samples were subsequently reverse-transcribed with the FIREScript^®^ RT cDNA synthesis kit (Solis Biodyne, Tartu, Estonia), following the manufacturer’s guidelines. The concentrations of the DNA/RNA and cDNA samples were measured with a NanoDrop^®^ ND-1000 UV-Vis Spectrophotometer (Waltham, MA, USA).

### 2.10. Quantification of Giant Viruses by Real-Time qPCR

Real-time qPCR was conducted using the SYBR green method, as previously described [26,27,28] on a Bio-Rad CFX 96 Real-Time PCR system (Bio-Rad Laboratories, Inc., Hercules, CA, USA) (Figures S1 and S2). Quantification was performed using the standard curve method, with calibration standards generated from viral DNA derived from ASFV-infected PAMs. The viral housekeeping gene K196R (thymidine kinase) was used as a reference gene for quantitative standardization. All samples were run in triplicate to ensure technical reproducibility, and non-template controls (NTCs) were included in each run to monitor for contamination. All samples were quantified against a standard curve of known genomic units. For alignment of the cDNA plots and infection titers of ASFV, Cq values were rescaled after comparing with viral genome copies and modified in absolute amounts along the *y*-axis for better visualization. Each 20 µL reaction mixture contained 4 µL of 5× HOT FIREPol^®^ EvaGreen^®^ RT-qPCR Mix Plus (ROX) (Solis BioDyne, Tartu, Estonia), 0.3 µL of each specific primer (initial concentration of 100 pmoL/µL), 4 µL of template cDNA, and 11.4 µL of ddH2O. The reaction conditions were as follows: polymerase activation at 95 °C for 12 min, followed by 40 cycles of 95 °C for 15 s, 52 °C for 30 s, and 72 °C for 30 s. Melt Curve Analysis was used under the following conditions: 65 °C to 95.0 °C; increment of 0.5 °C every 0:05 s. Primers used for amplification were designed based on ASFV Georgia 2007/1, for giant viruses, representatives of 4 families, including *Mimivirus, Phycodnavirus*, *Megavirus*, and *Marseillevirus*, and sequences in FASTA format and ordered from Integrated DNA Technology-IDT (https://eu.idtdna.com/, accessed on 11 May 2019). We selected EvaGreen^®^ chemistry (a next-generation saturating dye) for its ability to detect the broad genetic diversity inherent in ‘Marseille-like’ and ‘Faustovirus-like’ sequences. Probes require 100% sequence identity, which poses a risk of false negatives when investigating potentially novel lineages in insect vectors [26,28,29]. Furthermore, all our results were validated using Melt Curve Analysis, confirming the absence of non-specific artifacts and ensuring that our ‘microbiological demonstration’ meets the rigorous standards of the published literature in this field [30].

Primers are presented in Table 1.

### 2.11. Criteria of Inclusion and Exclusion for Mimivirus Presence

In the article, the inclusion and exclusion criteria were the detection of a positive qPCR for both studied genes simultaneously (only in this case, the primary criterion), microscopic examination on SEM (primary criterion), and microscopic detection of Gram-positive particles in the lung tissue of the consonant [2]—secondary criterion.

### 2.12. Statistical Analysis

In our work, we did not use a statistically sufficient sample, but cases of fatal viral pneumonia (*n* = 4) were tested for the presence of a number of viral pathogens of the human lungs, including mimivirus. Statistical tests were performed using SPSS (version 17.0; SPSS Inc., Chicago, IL, USA). For analytical assessment, the nonparametric Mann–Whitney U test was applied. Any differences were considered statistically significant if *p* < 0.05. Due to the very small number of samples, the Mann–Whitney U test was used to test the assumptions of normal distribution. As a nonparametric test based on ranks and providing accurate *p*-values, it offers a robust and statistically reliable alternative to the *t*-test for independent samples under these conditions [31,32].

## 3. Results

### 3.1. Clinics and Pathology

Clinical history and pathological/autopsy findings for all four patients are included in the Supplementary Materials under “Case Series”.

### 3.2. Mimivirus Detection by Real-Time qPCR

**Figure 1:**
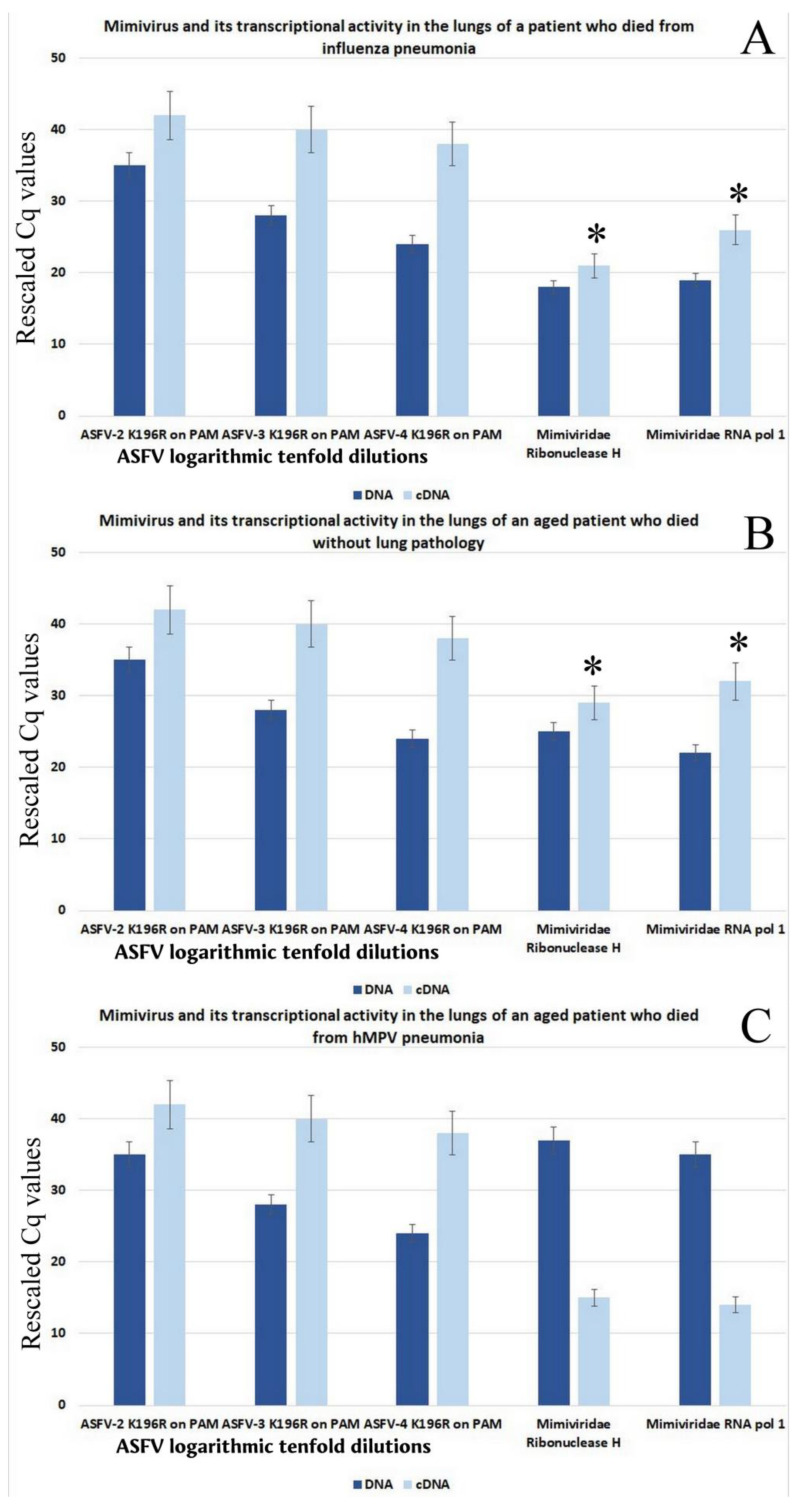
Presence and transcriptional activity of Mimivirus genes in human lung specimens at fatal viral pneumonias. (**A**) Presence of mimivirus and its transcriptional activity in fatal pneumonia caused by the H5N1 virus (age 52 years old). (**B**) Presence of mimivirus and its transcriptional activity in an elderly patient without pneumonia (age 93 years old). (**C**) Presence of mimivirus and its transcriptional activity in fatal pneumonia caused by hMPV (age 55 years). * significant (*p* < 0.05).

Standard real-time qPCR detection of mimivirus in lung tissue preceded quantitative analysis of the virus and viral gene transcription. As follows from Figure 1A, low levels of mimivirus were detected in a patient who died from influenza pneumonia, significantly lower than the maximum dilution of the ASF virus (lg −4), which corresponded to approximately 50 ASF virus particles/µL. However, the transcriptional activity of the virus was quite high—the level of transcripts significantly exceeded the level of the corresponding (reference) gene.

As shown in Figure 1B, in an elderly patient who died of causes other than pulmonary pathology, higher levels of mimivirus were detected, approximately identical to the maximum dilution of the ASF virus (lg −4), corresponding to approximately 50 ASF virus particles/μL. Despite the relatively high number of mimivirus genomes, its transcriptional activity was high—the level of transcripts significantly exceeded the level of the corresponding gene.

For comparison, data on the content and transcriptional activity of mitivirus genes in a patient who died from human metapneumovirus (hMPV) are presented in Figure 1C. The data indicate that the virus shows no transcriptional activity.

### 3.3. Visualization of Mimivirus-like Structures in Lung Tissue

**Figure 2:**
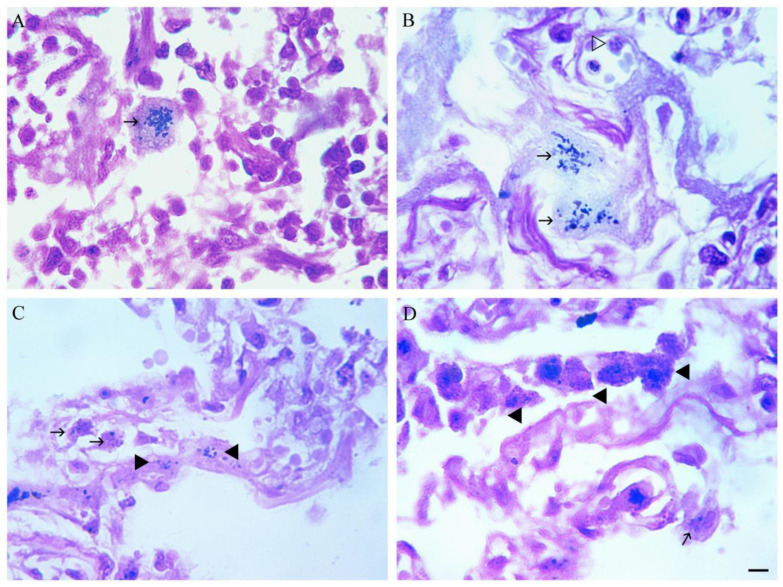
Gram staining of human lung tissue showing the presence of Gram-positive particles in macrophages. Gram staining and additional staining by picric acid. (**A**,**B**) Alveolar macrophages with Gram-positive particles in cytoplasm (arrowed). (**C**,**D**) Alveolar macrophages with Gram-positive particles in cytoplasm (arrowed); interstitial macrophages with Gram-positive particles in cytoplasm (triangle). Scale bar 10 µm.

Gram staining, according to Lusi et al. (2017) [2], revealed Mimivirus-like structures (MVLS) in both alveolar macrophages and pneumocytes (Figure 2).

As can be seen from Figure 2, Gram-positive particles (mimiviruses) are mainly detected in the cytoplasm of alveolar macrophages (Figure 2A–C). Sometimes, and in slightly smaller quantities, Gram-positive particles are also found in interstitial macrophages (Figure 2C,D).

### 3.4. Ncldv Assemblies (Possible Mimivirus) in Lung Tissue

**Figure 3:**
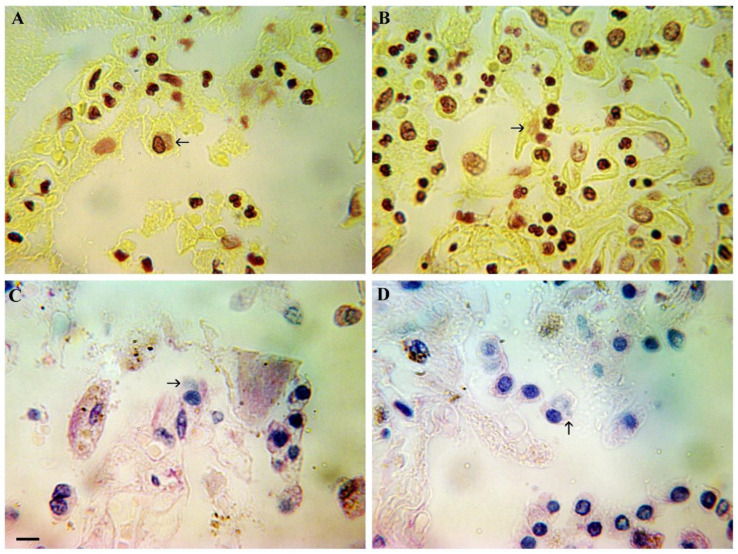
Detection of NCLDV assemblies in lung tissue. Scale bar 10 µm. (**A**) Feulgen–Naphthol Yellow S staining of lung tissue; an alveolar macrophage with a specific accumulation of DNA in the cytoplasm is visible (arrowed). (**B**) Feulgen–Naphthol Yellow S staining of lung tissue; an interstitial macrophage with a specific accumulation of DNA in the cytoplasm is visible (arrowed). (**C**) MGP staining of lung tissue; an alveolar macrophage with a specific accumulation of DNA in the cytoplasm is visible (arrowed). (**D**) MGP staining of lung tissue, an alveolar macrophage with a specific accumulation of DNA in the cytoplasm is visible (arrowed).

Detection of NCLDV-specific assemblies was performed using Feulgen–Naphthol Yellow S MGP stainings, which enable easy visualization of viral DNA clusters in the cytoplasm of infected cells. Staining by Feulgen–Naphthol Yellow S allows easy detection of viral DNA accumulations in the cytoplasm of NSFV-infected cells. As shown in Figure 3A, similar clusters are detected in the cytoplasm of alveolar macrophages. Sometimes, DNA clusters are detected in the cytoplasm of interstitial macrophages (Figure 3B). As a rule, the accumulation of viral DNA in the cytoplasm, when stained with Feulgen–Naphthol Yellow S, has a lighter shade compared to the staining of the cell nucleus, and lacks the characteristic and fairly specific chromatin structure visible under a microscope (Figure 3C,D).

MGP provides good contrast between all cellular structures and viral DNA assemblies. Methyl green binds preferentially and specifically to DNA (blue color), and pyronin binds RNA (pink or red color). It is important to note that all cells with DNA assemblies showed good visible color differentiation of NCLDV genome assemblies not only in the cytoplasm of infected cells, but also in the nuclei of infected cells. Viral DNA assemblies are colored in blue. But nuclear chromatin is colored blue, with reddish or greenish shades, according to the proportion of RNA contained in nuclei.

### 3.5. Lung Histopathology

**Figure 4:**
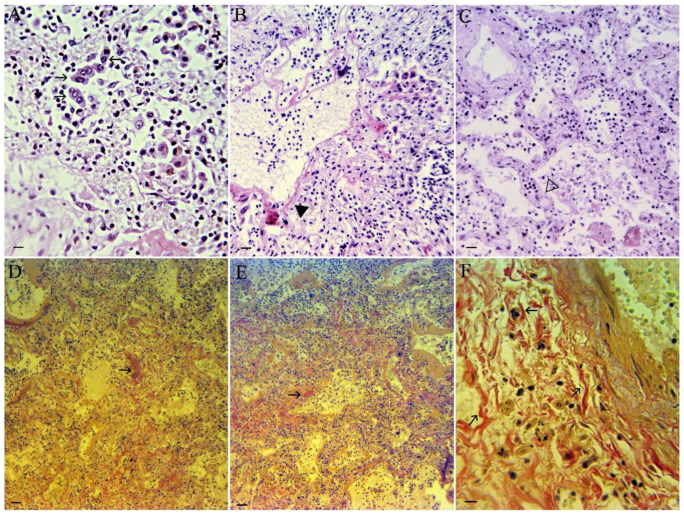
Histopathology of viral lung pneumonia. (**A**) Lumen of alveola with cellular infiltrates, where binuclear macrophages (arrowed) predominate, represented by both lymphocytes and polymorphonuclear leukocytes. Scale bar 10 µm. (**B**) Disorganized alveolar septa with hyalin membrane (black triangle); there is hypervascularization, but erythrocyte masses do not predominate due to cellular infiltrates. Scale bar 20 µm. (**C**) Thickened alveolar walls due to hyperemia and cellular infiltration (white triangle). Scale bar 20 µm. (**D**) Presence of collagen fibers in the lung with activated mimivirus (arrowed). Scale bar 30 µm. (**E**) Presence of collagen fibers in the lung with nonactive mimivirus (arrowed). Scale bar 30 µm. (**F**) Presence of separated collagen strands (arrowed) in the paravascular region of the lung with activated mimivirus. Scale bar 10 µm.

Histological examination revealed a standard picture of viral pneumonia at a late stage (Figure 4). Both the interalveolar spaces and the alveoli are filled with mixed cellular infiltrates: lymphoplasmacytic, neutrophilic, and macrophage (Figure 4A). Some macrophages are binucleated, the alveoli are fused due to disintegration of the interalveolar walls by diffuse alveolar damage (DAD), and there are foci of necrosis (Figure 4B). The bronchial lumen also contains abundant exudate mixed with remnants of exfoliated respiratory epithelium. The walls of the preserved alveoli are thickened due to hyperemia and cellular infiltration, and the lumen also contains cellular infiltrates (Figure 4C). Cellular infiltrates predominate, composed of both lymphocytes and polymorphonuclear leukocytes; alveolar septa are disorganized (DAD), and there is hypervascularization, but erythrocyte masses do not predominate. Alveolar walls are thickened due to hyperemia and cellular infiltration.

With Van Gieson’s stain (Figure 4D,E), newly formed collagen fibers are stained bright red, contrasting with the amorphous extracellular material, which is stained yellowish. In contrast, the elongated nuclei of fibroblasts, as well as other cell nuclei, are stained darkly. At higher magnification, the collagen masses are noticeably composed of separated collagen strands (Figure 4F).

**Figure 5:**
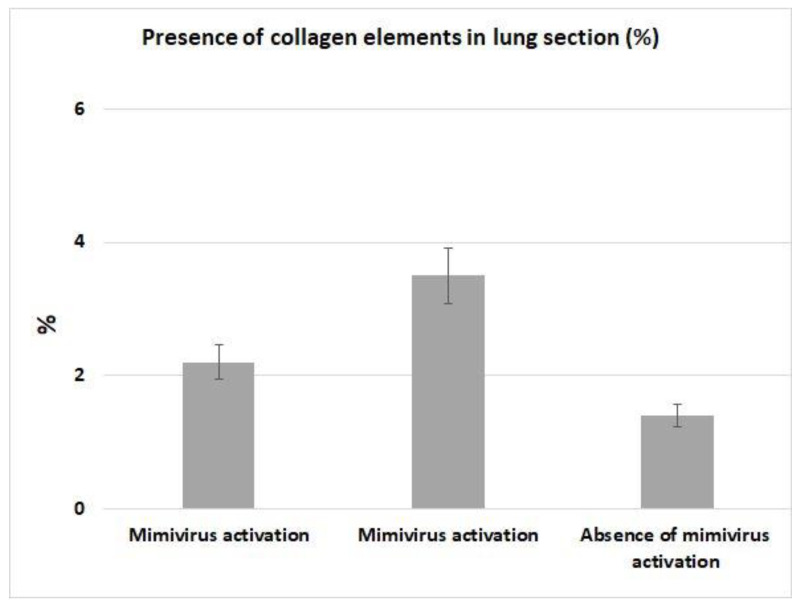
Presence of collagen elements in lung section.

Figure 5 presents data on lung collagen levels in patients who died from pulmonary pneumonia with mimivirus activation, compared with those who died from pulmonary pneumonia without mimivirus activation. As shown in Figure 5, in patients with mimivirus activation, more collagen fibers are detected in lung tissue. The measurements were conducted using digital analysis (Photopea software). However, it is impossible to conclude the resulting sample due to the insufficient number of cases.

### 3.6. Visualization of Mimivirus in Lung Tissue by SEM

**Figure 6:**
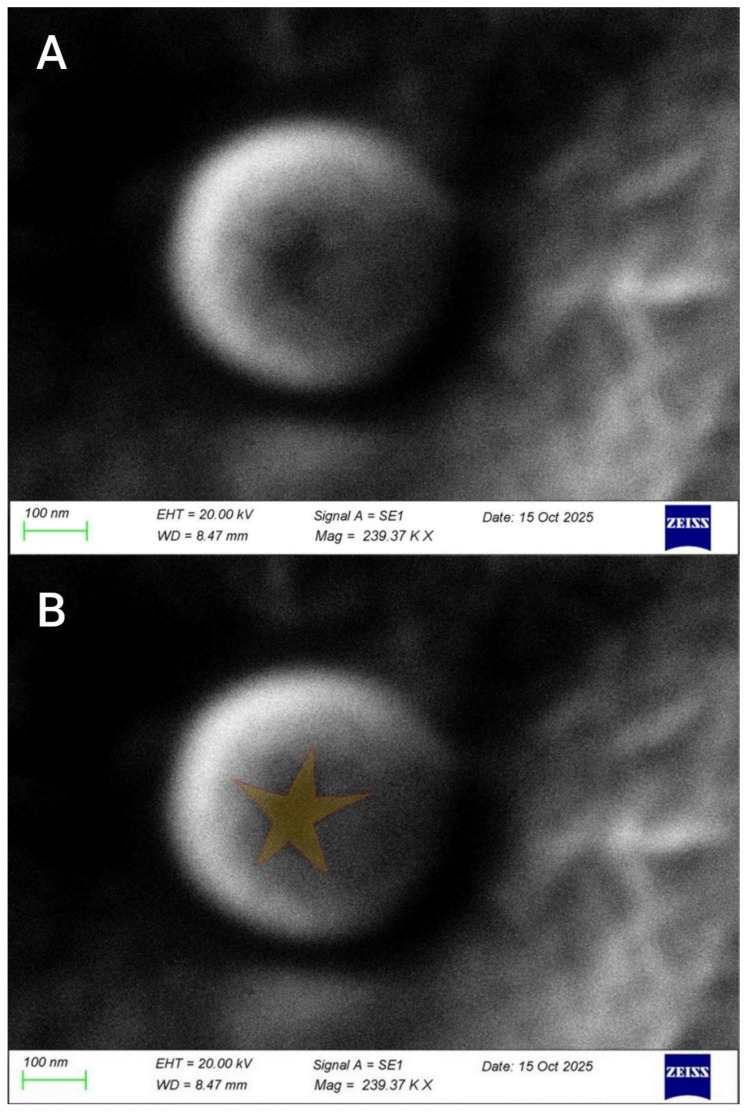
Mimivirus isolated post-mortem directly from lung samples. (**A**) Mimivirus-specific star-shaped structure. (**B**) Graphically supplemented star-shaped structure of mimivirus.

We were able to isolate and visualize Mimivirus directly from patient lungs post-mortem, without replication in amoebae or susceptible cell lines. Visualization was carried out using scanning electron microscopy. This is the first detection of mimivirus directly from human lung tissue, without prior in vitro replication. The mimivirus is shown in Figure 6.

As shown in Figure 5, we have identified viruses with a star-shaped structure within the capsid, a specific morphological characteristic of mimiviruses. The star structure is partially open at the capsid’s vertex. Also, it is important to note that the particles we detected are approximately 550–600 nm in size (Figure 6A), which corresponds to the size of the viruses reported in the work [4]. The ray span of the star-shaped structure is approximately 100 nm (Figure 6B).

## 4. Discussion

We have identified the presence of mimiviruses in patients with fatal forms of viral pneumonia. Validation of mimivirus was carried out with two primers, which is considered sufficient for confident [8]. Some authors believe that mimiviruses are rarely detected in human lungs (see, for example, [5]). This is most likely due to the discrepancy between the results obtained by serological and genomic methods. The latter, while undoubtedly much more accurate, produces false negative results due to the high polymorphism of nucleotide sequences in members of the Mimiviridae family [9,33].

Firstly, we found microscopically detectable NCLDV viral particles in lung autopsies that resemble mimivirus inclusion bodies found in vitro and are consistent with Gram-positive inclusion bodies observed in humans [2]. This indicates the replication of a large NCLDV in the cytoplasm of lung cells. Mimivirus is a fully cytoplasmic giant virus for which virions are internalized into the host cell by phagocytosis [34,35]. This explains why the virus is more frequently detected in the cytoplasm of alveolar macrophages than in pneumocytes. Viral DNA assemblies, stained in paler shades of blue-green (compared to nuclear structures when stained MGP) or paler pink (compared to nuclear structures when stained according to Feulgen), are clearly visible in the cytoplasm. The detection of such structures during histological examination of autopsy specimens also indicates NCLDV replication [17]. Despite the fairly frequent detection of mimivirus in lung tissue from patients who died of viral pneumonia, we cannot confirm or refute its role in the development of pulmonary pathology. While transcriptional activity of mimivirus genes has been reliably detected in patients who died from viral pneumonia, similar activity has also been detected in some elderly patients who died without any lung pathology. It should be added that viral assemblies specific for NCLDV are detected in lung tissues, where the transcriptional activity of mimivirus is demonstrated; however, the number of viral assemblies in lung tissue is insignificant, especially in comparison with other viruses that cause fatal pneumonia in humans, for example, in human metapneumovirus. Undoubtedly, the MGP staining is non-specific and can stain almost any giant NCLDV; however, it allows us to at least detect the replication activity of these viruses in tissues, as we have shown. Although it has previously been shown that mimivirus is capable of replicating in human blood mononuclear cells [35], we have demonstrated that mimivirus in human lungs is capable, if not of replicating, then of at least transcribing individual genes and forming viral assemblies in the cytoplasm. The susceptible cells are macrophages, primarily alveolar, but also interstitial to some extent. This confirms the conclusions [10] about both of the routes of virus penetration into the target cell through phagocytosis and the probable target cells—alveolar macrophages. In general, several amoebae pathogens, including mimiviruses, may be pathogenic to humans due to their resistance to human macrophages, especially mature cells, rather than monocytes [36,37].

It should be noted that we detected a high prevalence of mimivirus in lung tissue from patients with fatal viral pneumonia (three out of four cases, or 75%). Transcriptional activity of mimivirus genes in lung tissue was detected in two of three cases (67%). The presence of the virus is confirmed by both quantitative real-time qPCR and the detection of specific virus morphology under SEM. Possibly obtained data indicate the prognostic value of detecting mitivirus transcriptional activity in human lungs in viral pneumonia. Visualization of complete viral particles, together with detection of transcriptional activity, strongly indicates full-fledged viral replication in human lung tissue.

It is necessary to discuss the reasons for the relatively low mimivirus detection rate in patients with pneumonia, compared with our data [12,13,38,39]. One factor masking the presence of mimivirus in human lungs is likely its low abundance or absence in the bronchoalveolar lavage or respiratory secretions used by the authors of most of the cited articles. In our study, we analyzed lung tissue obtained at post-mortem autopsy, thereby enabling us to obtain higher quantities of viral particles. Another factor that allowed us to obtain a higher number of viral particles was post-mortem studies, during which the virus is at the terminal stage of the disease and can undergo more active replication.

Histological examination of a mouse model of mimivirus pneumonia revealed thickened alveolar walls with cellular infiltrates of mononuclear leukocytes, macrophages, and lymphocytes, as well as diffuse alveolar damage with formation of a hyaline membrane and erythrocytes in the alveolar lumen [38]. The authors reported the development of connective tissue elements during experimental infection with Mimivirus in mice. We also demonstrated an increase in areas containing connective tissue elements in lung sections from the same patients with mimivirus transcription, compared with lungs from patients without transcriptional activity of mimivirus. However, this characteristic is not specific enough to reliably link it to the development of mimivirus pneumonia. The histological characteristics of mimivirus pneumonia described by the authors are often seen in other viral pneumonias (see, for example, [40]).

We also did not detect any specific pathological changes in lung tissue associated with mimivirus infection. But despite the absence of specific pathology, the available data allow us to associate it with poor outcomes in some patients with viral pneumonia [41]. Our data indicate that severe forms of pneumonia may be associated not so much with the presence of mimiviruses in human lungs, but with the activation of transcription of the viral genome.

## 5. Conclusions

We have found a more widespread presence of mimiviruses in humans with fatal pneumonia than previously thought (probably due to the detection of viruses directly in lung tissue, rather than in BAL or in the upper respiratory tract). We have demonstrated transcriptional activity of mimiviruses in some patients with fatal pneumonia caused by other viruses (e.g., influenza virus). For the first time, a complete viral particle with a characteristic capsid structure has been isolated from human lung tissue. The data presented above suggest that it is not so much the presence of mimiviruses in human lungs, but rather the activation of transcription of the viral genome that may be associated with severe forms of pneumonia.

## Figures and Tables

**Table 1:** Primers used for real-time qPCR.

Gene	Sequence (5′-3′)
Thymidine kinase K196R	F: GCAGTTGTCGTAGATGAAG R: CGAAGGAAGCATTGAGTC
Nucleoprotein (N) hMPV	F: GAGTCTCAGTACACAATAA R: GCATTTCCGAGAACAACAC
Mimivirus BAV	F: CAGATTCTACTTACAGTGTCAATA R: GACCAGTATGTGCTTCAAC
Mimivirus R651	F: CAGAATCAATCACCGAATC R: CCAATAGTATCAACAGAATCAT

## Data Availability

The original contributions presented in this study are included in the article/Supplementary Materials. Further inquiries can be directed to the corresponding author.

## Supplementary Materials

The following supporting information can be downloaded at: https://www.mdpi.com/article/10.3390/v18030379/s1: Figure S1: Standard Curve calibration plot of real-time qPCR; Figure S2: The log dilutions (−1, −2, −3) of ASFV-infected PAM cells with the K196R gene.
