# Supplementary material for: Post-Mortem Detection and Visualization of Mimivirus Reactivation in Fatal Viral Pneumonia
Source: Viruses. 2026 Mar 18;18(3):379. doi: 10.3390/v18030379 (PMC13030392; doi:10.3390/v18030379)
Supplement: Supplementary file 1 [file viruses-18-00379-s001.zip › Case series-Viral pneumonia.pdf]

Case series: Fatal Viral Pneumonia

Patient 1 (84) Mimivirus genome absent

Clinical History

The 52 year-old male patient was admitted to the hospital on the 29th of April, 2025 with severe general weakness, shortness of breath, and dyspnea. According to the patient's family, his symptoms had started 2 days earlier with a fever and chills. The patient's comorbidities included diabetes mellitus type 2 and secondary arterial hypertension. Upon admittance, the patient was conscious, with pallor/cyanosis of the skin and visible mucous membranes. His breathing was tachypneic, shallow, and asthmatic with prolonged exhalation time. The patient also had decreased oxygen saturation (SpO_2- 80%), for which inhalation therapy was administered. Echocardiography revealed a cardiac ejection fraction of 50%, III°-IV° mitral regurgitation, II°- III° tricuspid regurgitation, LV anterolateral wall akinesia, and possible prolapse of the anterior leaflet of the mitral valve, likely due to papillary muscle tearing. In the emergency department the patient received oxygen-therapy, infusion-therapy, antibiotic therapy, insulin therapy, gastroprotectants, anticoagulants, antiaggregants, ionotropic drugs, diuretics, and symptomatic treatment. Laboratory analysis confirmed the presence of hMPV by Real-time qPCR .

The patient's condition rapidly worsened that night with increasing respiratory insufficiency (SpO_2- 65%), and the patient was intubated and placed on artificial ventilation. A multi-organ CT scan was conducted on 30th of April which revealed bilateral polysegmental pneumonia, pulmonary edema, and bilateral hydrothorax (after drainage of the right pleural cavity). On the 5th of May, there was some improvement in the patient's bilateral polysegmental pneumonia, decreased pulmonary edema, and minimal bilateral hydrothorax. However, by the 12th of May, the bilateral polysegmental pneumonia, pulmonary edema, and bilateral hydrothorax had worsened again. On the 19th of May, the ECG monitor recorded bradycardia and eventually asystole. After 30 minutes of unsuccessful CPR, the patient was pronounced dead.

Pathological/Autopsy Findings

Postmortem studies determined that the main diseases were: bilateral polysegmental pneumonia accompanied by bilateral exudative pleuritis; repeated acute myocardial infarction of LV anterolateral wall; diabetes mellitus type 2 accompanied by diabetic nephropathy, micro- and macroangiopathy; generalized atherosclerosis accompanied by atherosclerotic stenosis of coronary arteries (III°); postinfarction cardiosclerosis; concentric hypertrophy of the left ventricle.

Complications of the diseases included acute generalized microcirculatory hemodynamic disturbances, multiple micro-hemorrhages of the serosal and mucosal surfaces, pulmonary edema with brown induration of the lungs, severe hepatic and myocardial parenchymal degeneration with areas of necrosis, acute necronephrosis, and hydrothorax.The underlying causes of death were determined to be bilateral polysegmental pneumonia and repeated acute myocardial infarction, while the immediate cause of death was mixed-shock with multiple organ failure.

Patient 2 (13) Mimivirus genome present transcription absent

Clinical History

The 55-year-old male patient was hospitalized, complaining of shortness of breath and swelling in the testicular and paratesticular area. Concomitant diseases include liver cirrhosis, diabetes mellitus for 4–5 years, rib resection, and lung abscess drainage (5 years ago).

At admission on 24 November, consciousness was clear, the skin and visible mucous membranes were yellowish, and the limbs were slightly swollen with complaints of a slight fever, headache, and muscle aches. Symptoms like cough, sore or dry throat, nasal congestion, and nausea were not manifested. White blood cell count was 20.53 × 109/L, red blood cells 3.27 × 1012/L, and C-reactive protein was 115.34 mg/L (peak value). (Table S1) Total bilirubin 102.5 μmol/L, direct bilirubin 72.7 μmol/L, creatinine- 137 μmol/L, Gamma-glutamyl transferase 161.5 U/L, total protein 61.1 g/L, albumin 23.25 g/L, prothrombin time 34.8″, international normalized ratio 2.73. An X-ray revealed subsegmental bilateral infiltration. CT scan showed moderate pleural effusion on both sides, diffuse interlobular septal thickening, and a postoperative cavity in the upper lobe of the right lung (Figure 1A,B). No lymphadenopathy was detected. Treatment with antibiotics was started. Treatment with Azithromycin 500 mg once a day was started because of persistent fever for 5 days. Additionally, hepatoprotectors and gastroprotectors, antidiuretics, and infusion therapy and were prescribed. Normal Saline and 20% Albumine were used with Spirinolactone as an antidiuretic for infusion therapy to manage the edema. Omeprazole and Sucralfate were added for gastroprotection. Essentiale forte was the hepatoprotective drug. The dosages were strictly controlled, and laboratory parameters were strongly monitored (especially Na^+, K^+, Mg^{2+}, glucose, creatinine, etc.).

Due to the worsening of the situation with increasing temperature, surgical intervention was performed on 13 December: orchiectomy from the right side with excision of Fournier's gangrene. The patient recovered smoothly after surgery, with some positive dynamic changes in the laboratory parameters, but the shortness of breath and weakness remained. On 7 January, the patient was urgently transferred to the intensive care unit with critical shortness of breath, cough, and hemoptysis.CT observation less than 4 days before death revealed minimal pleural fluid on both sides (blue arrows) up to 0.8 cm thick (insignificant); also, on both sides, mostly on the left in the basal sections, minimally consolidated zones were observed (orange arrows) (Figure 1C,D). No pneumothorax was observed; increased thickening of interlobular septa in the lungs was observed, and postoperative cavity in the upper lobe of the right lung was 3.8 cm. The hardened zone in the pleural region of the 2nd–3rd intercostal space on the right 3.3 × 3.0 cm—probable postoperative—was without dynamics. No lymphadenopathy was detected. The lumens of the trachea and main bronchi were free.

Saturation progressively decreased during the next three days despite treatment, and wet crackles were auscultated. Due to the severe condition of the patient, additional CT scanning for the last four days was impossible. On 11 January, the patient died. In fact, the patient developed an acute form of viral pneumonia with typical symptoms of fever, cough, runny nose, chest pain, and headache. It is necessary to note the relative speed of pneumonia development: from the full development of symptoms to death, less than 96 h passed.

The autopsy revealed bilateral hemorrhagic pneumonia with lymphadenopathy. Lungs were totally heavy and congested, with subpleural hemorrhages, hemorrhagic and edematous tracheal and bronchial mucosa, and diffuse hemorrhagic consolidations on the cut surface.

Laboratory testing

Laboratory testing was negative for autoimmune disease, including normal IgG and IgM cardiolipin antibodies, normal Beta-2-Glycoprotein IgG and IgM antibodies, negative antinuclear antibodies, negative antineutrophil cytoplasmic antibodies, negative rheumatoid factor, and negative antiglomerular basement membrane antibody. Infection diagnostic tests included negative HIV-1/2 antibodies and HIV-1 antigen, negative Legionella and pneumococcal antigens, negative lung Herpesviridae (Herpes simplex virus 1; Herpes simplex virus 2; Epstein–Barr, cytomegalovirus); negative SARS-CoV-2 (measured by quantitative reverse transcription polymerase chain reaction (qRT-PCR); negative hepatitis viral panel, negative

Aspergillus galactomannan antigen and galactomannan index, negative mycoplasma, negative dengue virus presence (procedure related to imported cases of Dengue fever in tourists from Armenia, measured by qRT-PCR). The influenza virus was excluded while the patient was still alive in the clinic, and its absence was confirmed postmortem.
Laboratory analysis confirmed the presence of hMPV by Real-time qPCR .

Pathological Findings

Postmortem examination of the lungs starts six hours after death. Cut sections from lungs stained with routine hematoxylin–eosin revealed edema, hemorrhages in intra- and interalveolar spaces, with focal rupture of interalveolar septae and interstitial lymphocytic infiltration (Figure 3A). Postmortem lung aspirate revealed large numbers of neutrophils, lymphocytes, erythrocytes and epithelial cells (predominantly airway mucosa).

Lung impression smears prepared routinely [7] and stained both with Pappenheim and with hematoxylin and eosin. Multinucleated cells, predominantly of macrophage origin, are easily identified on the impression smears (Figure 3B). A significant proportion of pulmonary macrophages show signs of autophagy (Figure 3C), which is an important part of pulmonary pathology [8]. Lung impression smears also revealed a high number of leukocytes, primarily neutrophils (Figure 3D). Histological studies revealed an eosinophilic inclusions in the cytoplasm of epithelial cells, which are characteristic of mononegaviral pathology, and in particular hMPV (Figure 3E,F). Compared to other mononegaviruses, eosinophilic inclusions found in alveolocytes are quite large, occur frequently and are easily detected.

Patient 3 (26) Mimivirus genome present transcription present

Clinical History

The 68 year-old male patient was hospitalized January 31th, 2025 with severe general weakness, shortness of breath, and a rise in body temperature. 5 days earlier, the patient reported having had a fever (38.5°C), shortness of breath, dyspnea, and diffuse dry wheezing/crackles. The patient complained of numbness and stiffness of the lower extremities. On the day of admittance the patient had noticed impaired consciousness before going to the hospital. According to the patient's family, the patient had considered himself ill for many years. In 2012, the patient had a stent placement after myocardial infarction. In 2020, the patient underwent coronary artery bypass surgery. The patient suffered from concomitant arterial hypertension and diabetes mellitus type 2 (for the last 10 years). The patient was previously hospitalized with acute respiratory insufficiency on December 30th, 2024. He was subsequently transferred to the cardiology department and eventually released from the hospital on January 9th, 2025.

Upon admittance, the patient tested negative for SARS-Cov2, and positive for influenza A. The patient's general condition was evaluated as extremely severe. The patient's exhibited clear consciousness (15 on the Glasgow coma scale), independent breathing, decreased vesicular breathing upon auscultation, and diffuse wet crackles. BP was 130/98, heart rate was 88 beats/minute. The patient also had decreased oxygen saturation (SpO_2- 51%-77%), and was administered oxygen. ECG revealed left axis deviation and LV hypertrophy. Echocardiography revealed a cardiac ejection fraction of 40%, mitral regurgitation II°, tricuspid regurgitation II°, dilated left atrium, concentric hypertrophy of the left ventricle, and mildly decreased left ventricular contractility. Troponin was 93 pg/mL.

Chest X-ray on the right side revealed subsegmental infiltrates in the perihilar and middle zones. The left side showed decreased translucency in the perihilar, middle and lower zones. There were also signs

of interstitial edema, hilar homogenization. There is also expansion of the upper-middle mediastinum especially on the right side, and thickening of the pleura. Meanwhile, abdominal ultrasound reveals enlarged liver, without diffuse parenchymal changes, homogenous. Gallbladder is shortened The pancreas shows diffuse changes and is homogenous. The spleen is 15cm. Kidney size was unchanged, with dilation of the pelvicalyceal system and disruption of urine outflow. CT scanning on February 4th showed bilateral polysegmental atypical pneumonia- 80-85%, and cardiomegaly.

In the emergency department the patient received oxygen-therapy, infusion/transfusion therapy, antibiotic therapy, gastroprotectants, ionotropic drugs, and symptomatic treatment.

On February 2nd, as a result of respiratory insufficiency the patient was put on artificial ventilation through a CPAP mask. On February 3th, the patient was intubated and placed on invasive artificial ventilation. On February 6th, the patient's condition rapidly worsened and the ECG monitor recorded bradycardia which rapidly progressed to asystole. CPR was immediately performed, along with indirect cardiac massage, injections of atropine and adrenaline. After 12 minutes the patient's cardio monitor revealed ventricular tachycardia, which was followed by defibrillation and restoration to sinus rhythm. On the February 7nd, the ECG monitor recorded bradycardia progressing to asystole. After 20 minutes of CPR, the patient was successfully resuscitated: Later that day the cardiac monitor recorded bradycardia which rapidly progressed to asystole. After 40 mins of unsuccessful CPR, the patient was pronounced dead.

Pathological/Autopsy Findings

Postmortem studies determined that the main diseases were: bilateral polysegmental pneumonia accompanied by bilateral exudative pleuritis; acute subendocardial myocardial infarction of LV anterior wall; diabetes mellitus type 2 accompanied by diabetic micro- and macroangiopathy; generalized atherosclerosis accompanied by atherosclerosis of aorta (IV°) and atherosclerotic stenosis of coronary arteries (III°); concentric hypertrophy of the left ventricle.

Complications of the diseases included acute generalized microcirculatory hemodynamic disturbances, multiple micro-hemorrhages of the serosal and mucosal surfaces, pulmonary edema with brown induration of the lungs, bilateral hydrothorax, severe myocardial parenchymal degeneration with necrotic foci, acute necronephrosis. The underlying causes of death were determined to be bilateral polysegmental pneumonia and acute subendocardial myocardial infarction, while the immediate cause of death was mixed-shock.

Patient 4 (16) Mimivirus genome present transcription present

Clinical History

The 93 year-old female patient was hospitalized January 12th, 2025 in critical condition, and unable to state her symptoms. The patient's family reported that symptoms started a day earlier, and upon worsening, they brought her to the hospital. The patient had previously undergone a cholecystectomy, a surgery related to a periauricular benign tumor, as well as colectomy and formation of anastomosis due to a malignant neoplasm. The patient was admitted with a Glasgow Coma Scale score of 13-14 in consciousness, pallor of skin and visible mucous membranes, and independent breathing with nasal cannula. Her blood pressure was 149/74 mmHg, heart rate 104 beats/min., and a temperature of 37°C. CT scan revealed bilateral lower lobar pneumonia, and bilateral moderate pleural effusion, severe cardiomegaly, prominent pulmonary edema, and pulmonary hypertension. The patient received oxygen-therapy, infusion-therapy, antibiotic therapy, gastroprotectants, water/electrolyte balance correction, and symptomatic treatment.

Laboratory analysis did not detect any respiratory infection by Real-time qPCR.

On January 21,2025 the patient's condition rapidly declined. Bradycardia was noted, quickly followed by asystole and drop in blood pressure. The patient was immediately administered CPR, indirect heart massage, drug therapy and artificial ventilation. After 30 minutes of unsuccessful resuscitation, the patient was pronounced dead.

Pathological/Autopsy Findings

Postmortem studies determined that the main diseases were: bilateral polysegmental pneumonia accompanied by bilateral exudative pleuritis; acute subendocardial myocardial infarction of LV anterior wall; generalized atherosclerosis accompanied by atherosclerosis of aorta (IV°) and atherosclerotic stenosis of coronary arteries (III°); concentric hypertrophy of the left ventricle.

Complications of the diseases included acute generalized microcirculatory hemodynamic disturbances, multiple micro-hemorrhages of the serosal and mucosal surfaces, pulmonary edema with brown induration of the lungs, acute necronephrosis, and hydrothorax. The underlying causes of death were determined to be bilateral polysegmental pneumonia and acute subendocardial myocardial infarction, while the immediate cause of death was mixed-shock.

Patient 1 - AM 84, 52 y.o. male hMPV and bacterial component

Patient 2 - AM 13, 55 y.o. male hMPV

Patient 3 - AM 26, 68 y.o. male H5N1

Patient 4 - AM 16, 93 y.o. female; mainly age-related changes in lungs

	Patient 1	Patient 2	Patient 3	Patient 4
Age	52	55	68	93
Gender	male	male	male	female
Days from onset to hospitalization	2	-	5	1
Days from hospitalization to death	20	48	8	9
**Symptoms**				
Rise in temperature	yes	yes	yes	yes
Chills	yes	no	no	no
Weakness	yes	yes	yes	-
Shortness of breath	yes	yes	yes	-
Dyspnea	yes	no	yes	no
Pallor/cyanosis	yes	no	no	yes
Disturbances in consciousness	no	no	yes	yes
Cough	no	yes	no	no
Hemoptysis	no	yes	no	no
**Complications**				
Shock	yes	yes	yes	yes
Hemodynamic disturbances	yes	yes	yes	yes
Hepatic parenchymal degeneration	yes	-	yes	no
Myocardial parenchymal degeneration	yes	-	yes	yes
hydrothorax	yes	yes	yes	yes
Brown induration of lungs	yes	yes	yes	yes
Multiple organ failure	yes	-	no	no
**Treatment**				
oxygen-therapy	yes	yes	yes	yes
Infusion-therapy	yes	yes	yes	yes
gastroprotectants	yes	yes	yes	yes
Artificial ventilation	yes	yes	yes	yes
