# Supplementary figures and images for: Post-Mortem Detection and Visualization of Mimivirus Reactivation in Fatal Viral Pneumonia
Source: Viruses. 2026 Mar 18;18(3):379. doi: 10.3390/v18030379 (PMC13030392; doi:10.3390/v18030379)

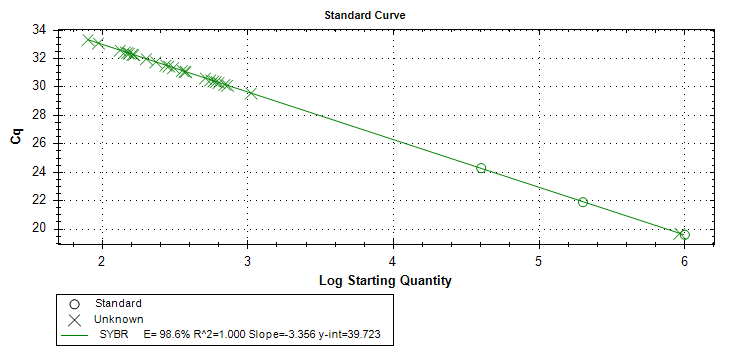

Supplement: Supplementary file 1 [file viruses-18-00379-s001.zip › Fig S1.png]

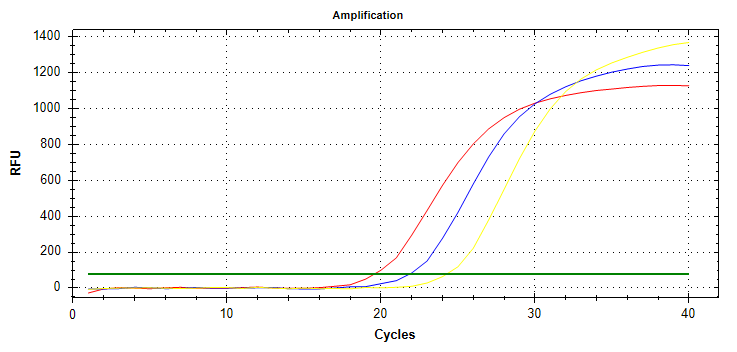

Supplement: Supplementary file 1 [file viruses-18-00379-s001.zip › Fig S2.png]
